# Synthesis of Solketal Catalyzed by Acid-Modified Pyrolytic Carbon Black from Waste Tires

**DOI:** 10.3390/molecules29174102

**Published:** 2024-08-29

**Authors:** Jolanta Kowalska-Kuś, Anna Malaika, Agnieszka Held, Aldona Jankowska, Ewa Janiszewska, Michał Zieliński, Krystyna Nowińska, Stanisław Kowalak, Klaudia Końska, Krzysztof Wróblewski

**Affiliations:** 1 Faculty of Chemistry, Adam Mickiewicz University, Uniwersytetu Poznańskiego 8, 61-614 Poznań, Poland; anna.malaika@amu.edu.pl (A.M.); awaclaw@amu.edu.pl (A.H.); aljan@amu.edu.pl (A.J.); eszym@amu.edu.pl (E.J.); mardok@amu.edu.pl (M.Z.); krysnow@amu.edu.pl (K.N.); 2 Contec, al. Jerozolimskie 142A, 02-305 Warszawa, Poland; k.konska@contec.tech (K.K.); k.wroblewski@contec.tech (K.W.)

**Keywords:** recovered carbon black, waste tire recycling, glycerol acetalization, solketal, carbon functionalization by H_2_SO_4_ and BDS

## Abstract

Solketal, a widely used glycerol-derived solvent, can be efficiently synthesized through heterogeneous catalysis, thus avoiding the significant product losses typically encountered with aqueous work-up in homogeneous catalysis. This study explores the catalytic synthesis of solketal using solid acid catalysts derived from recovered carbon blacks (rCBs), which are obtained through the pyrolysis of end-of-life tires. This was further converted into solid acid catalysts through the introduction of acidic functional groups using concentrated H_2_SO_4_ or 4-benzenediazonium sulfonate (BDS) as sulfonating agents. Additionally, post-pyrolytic rCB treated with glucose and subsequently sulfonated with sulfuric acid was also prepared. Comprehensive characterization of the initial and modified rCBs was performed using techniques such as elemental analysis, powder X-ray diffraction, thermogravimetric analysis, a back titration method, and both scanning and transmission electron microscopy, along with X-ray photoelectron spectroscopy. The catalytic performance of these samples was evaluated through the batch mode glycerol acetalization to produce solketal. The modified rCBs exhibited substantial catalytic activity, achieving high glycerol conversions (approximately 90%) and high solketal selectivity (around 95%) within 30 min at 40 °C. This notable activity was attributed to the presence of -SO_3_H groups on the surface of the functionalized rCBs. Reusability tests indicated that only rCBs modified with glucose demonstrated acceptable catalytic stability in subsequent acetalization cycles. The findings underscore the potential of utilizing end-of-life tires to produce effective acid catalysts for glycerol valorization processes.

## 1. Introduction

The continuous development of road transport worldwide has led to a steady increase in waste tires. According to Sofi [1], one billion tires are disposed of each year. A significant number of these waste tires are still stored in landfills, contributing to soil, water, and air pollution [2,3]. The shape of tires further exacerbates the problem by promoting water retention and contamination. Utilizing waste tires is a complex process due to their multi-component structure, and several methods are available. These include burning in cement kilns [4,5] and converting them for energy production, although these methods generate significant pollutants. Among various utilization methods—such as retreading, energy recovery, pyrolysis, and recycling—pyrolysis is the most versatile technology. It produces syngas, oil, and recovered carbon black (rCB). The efficiency of waste tire processing via pyrolysis depends on several factors, including pyrolysis temperature, reactor structure, total filler amount, presence of impurities, inert gas flow, and ash content [6,7]. The use of catalysts also plays a crucial role in the pyrolysis process, affecting the ratio of gas products, tire pyrolysis oil (TPO), and recovered carbon black [7].

Recently, extensive research has been conducted on the composition and application of pyrolysis products. Traditional carbon black (CB) is an industrial product, obtained by carbonization of hydrocarbon fuels, such as coal, tar, or gas, at relatively high temperatures. The resulting carbon black contains 95–99% pure carbon. It is characterized by a low content of mineral substances (max. 0.5%) and sulfur (max. 1%), with virtually no volatile or rubbery substances observed. In contrast, recovery carbon black (rCB) obtained from the pyrolysis of car tires contains less carbon compared to traditional carbon black (up to 80%). rCB is contaminated with a significant amount of mineral compounds (16–22%). It usually contains volatile substances and resins (1%), sulfur (4–5%), and zinc (5–7%) [8]. According to [7], depending on the composition of waste tires and the conditions of the pyrolysis process, the solid product, rCB, can be used as an adsorbent for separating gas and liquid mixtures, including organic compounds and dyes [9,10]. Moreover, after modification with acidic or basic reagents, rCB can be utilized as a catalyst for various processes [11] and as an anode material in alkali metal (Na or Li) batteries [12,13,14]. Carbon materials from waste tire pyrolysis have been used as catalysts for the catalytic cracking of municipal solid waste (MSW) and refuse-derived fuel (RDF), resulting in high hydrogen production [15]. The significant activity of rCB in the cracking process is due to the metal ions present in the material. It has been indicated that metal oxides or metal particles such as Cu, Fe, Ni, and Co show substantial activity not only for MSW and RDF cracking but also for the hydrogenation of bio-oil [11,16].

Recently, a number of authors have also indicated the high potential of carbon materials originating from carbohydrates and also biological wastes, after modification with sulfuric acid or alkaline modifiers, as catalysts for glycerol ketalization [17,18,19] and other acid-catalyzed processes [20,21]. However, recovered carbon materials obtained from the pyrolysis of waste tires have been applied to a relatively low extent as catalysts. Ayoob et al. [22] indicated that waste tire-derived activated carbon (WTAC), modified with KOH, may be successfully applied for biodiesel production based on the transesterification of non-edible oils with methanol or ethanol. On the other hand, we have previously indicated that pyrolytic residue from waste tires, modified with sulfuric acid, shows high and stable activity for the glycerol acetylation reaction [23]. However, although some applications of rCB materials as catalysts are indicated in the literature, the use of recovered carbon materials derived from the pyrolysis of waste tires as potential catalysts remains limited [11,16].

One of the intriguing possibilities for using acid-carbon catalysts derived from waste tires is their application in glycerol valorization, a by-product of biodiesel synthesis, an alternative fuel similar to conventional diesel. The high production cost is a major barrier to the widespread use of biodiesel as a substitute for petroleum diesel [24]. To address this challenge, various strategies have been explored and are currently under investigation to reduce biodiesel production costs, making it a more competitive fuel. These strategies include using highly active catalyst alternatives [25,26,27,28], as well as adopting technologies that require minimal energy input and enable faster transesterification. To further enhance the viability of biodiesel synthesis, efforts have been made to effectively utilize the excess waste glycerol [29,30,31] and to search for catalysts that are active in crude glycerol processing, including the ketalization of glycerol to solketal [29]. Among the different proposals, the application of rCB in the acetalization of glycerol to produce solketal seems to be particularly attractive [32,33]. This process not only aids in the effective management of glycerol by-products but also generates a valuable chemical compound with significant industrial applications. Solketal, the product of glycerol transformation with acetone, serves as a fuel additive, improving fuel properties and contributing to more sustainable energy solutions. According to Corrêa et al. [17], solketal enhances the octane number and oxidation stability of fuels while also reducing gum formation. Additionally, due to its high miscibility, solketal can be applied as a solvent for resins, paints, printing inks, and cleaning agents. Furthermore, its low toxicity makes solketal suitable for various pharmaceutical and cosmetic applications.

Conventionally, the condensation of glycerol with acetone is performed using large amounts of strong homogeneous Brønsted acid catalysts [34,35]. However, these methods have limitations, such as the use of unrecyclable and expensive reagents, and the need to neutralize the strongly acidic media, leading to the production of undesired wastes. The sustainability of the process can be improved by substituting homogeneous catalysts with heterogeneous ones. So far, several heterogeneous catalysts have been reported to be active in the condensation of glycerol with acetone. These include Amberlyst resins [36], sulfonic mesostructured silicas [37], modified silicalite with zeolite structure [38], and silica-supported heteropolyacids [39]. Among the explored catalysts, also zeolites demonstrated special potential, as highlighted in our previous studies, due to their cost-effective, large-scale production [29,40,41]. The activity of these catalysts is generally attributed to their Brønsted acidity [42,43,44,45].

Taking into account the above, in this paper, we explored the use of pyrolytic rCB materials, after acidic modification, as heterogeneous catalysts for transforming glycerol, a by-product of biodiesel production, into solketal. The idea behind using rCB, an industrial waste material produced from end-of-life tires by means of the so-called Molten technology, was to explore its potential in the role of a catalyst for producing valuable compounds such as solketal. An additional advantage of the research is the possibility of using glycerol derived from the biodiesel production process. Thus, the utilization of two potential industrial waste products (rCB and glycerol) adds value to the ongoing process. Our previous experience with the use of acidified rCBs in the glycerol acetylation process indicated the potential of this material. Additionally, to the best of our knowledge, this is the first time that such pyrolytic materials have been utilized in this capacity. Although carbon materials have demonstrated promise in other catalytic processes [15,17,18,19,29,41], their application in solketal production is less explored. The transformation of glycerol to solketal has only been performed on acidified CB produced from clean biological materials, mainly sugars [46]. Therefore, this work aimed to evaluate the method of generation of acid sites in pyrolytic rCB materials, optimize reaction conditions (time and temperature), and assess the reusability of these novel acidic waste tire-based catalysts. For this purpose, two different methods of acid site generation were employed: sulfuric acid treatment and using 4-benzenediazonium sulfonate (4-BDS). Additionally, glucose-pretreated rCB materials were obtained, resulting in new composite materials, which were then sulfonated to increase the reusability of the tested catalysts. The initial rCB, as well as the obtained acid–carbon materials, were characterized by elemental analysis, X-ray photoelectron spectroscopy (XPS), powder X-ray diffraction (PXRD), scanning electron microscopy (SEM), transmission electron microscopy (TEM), Fourier transform infrared (FT-IR) spectroscopy, and thermal gravimetric analysis (TGA).

## 2. Results and Discussion

### 2.1. Physicochemical Characterization of the rCB Samples

The rCB (recovered carbon black) material, obtained from the pyrolysis of waste tires at the CONTEC company, was modified with sulfuric acid (SA) or 4-benzenediazonium sulfonate (BDS) to induce acidic properties for subsequent use as catalysts in glycerol acetalization reaction with acetone. Additionally, the introduction of glucose followed by incomplete carbonization and sulfonation procedures was applied to improve the stability of the obtained catalysts.

**Figure 1:**
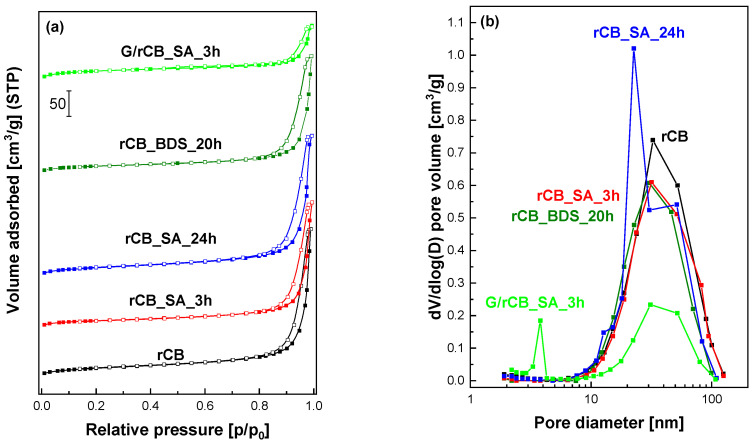
The influence of applied modification on the porosity of rCB samples: (**a**) nitrogen adsorption–desorption isotherms, (**b**) pore size distributions.

The results of the textural analysis of rCB samples are presented in Table 1. Figure 1a,b show the nitrogen adsorption/desorption isotherms and pore size distributions, respectively. According to the new IUPAC classification and based on the analysis of the adsorption/desorption isotherms depicted in Figure 1a, it can be inferred that rCB and modified materials exhibit a type IV isotherm with H2 hysteresis loop, which is characteristic of mesoporous adsorbents [47]. 

The specific surface area of rCB samples was influenced by the type of sulfonating agent, the duration of the modification, and the use of glucose during the preparation step (please see Table 1). The treatment of rCB sample with sulfuric acid or 4-benzenediazonium sulfonate only slightly affects its specific surface area and pore size distribution. When sulfuric acid was used, a short sulfonation time of 3 h reduced the BET specific surface area (S_BET_) of rCB material from 75 m^2^/g to 68 m^2^/g for the rCB_SA_3h material. In contrast, extending the sulfonation time to 24 h increased the BET surface area from 75 m^2^/g to 91 m^2^/g, with an almost unchanged porous structure. Additionally, the increase in the micropore surface area (S_micro_) and micropore volume (V_micro_) were observed for these materials. The use of BDS results in significant decreasing in specific surface area, pore volume and average pore diameter (Table 1). It is therefore reasonable to conclude that the Ph-SO_3_H groups on the carbon surface occupied part of the porous space, leading to a noticeable reduction in porosity. However, the mesopores of the carbon materials were not severely blocked by these groups. Similar observations have been made by other authors [48]. Incorporation of glucose via the wet impregnation method, followed by carbonization and sulfonation, significantly altered the specific surface area and affected the porosity. The surface area increased from 75 m^2^/g to 111 m^2^/g compared to the initial material, and S_micro_ increased from 3 m^2^/g to 67 m^2^/g. This is particularly evident in the BJH pore size distributions (Figure 1b), which show the presence of pores approximately 3 nm in size. 

**Figure 2:**
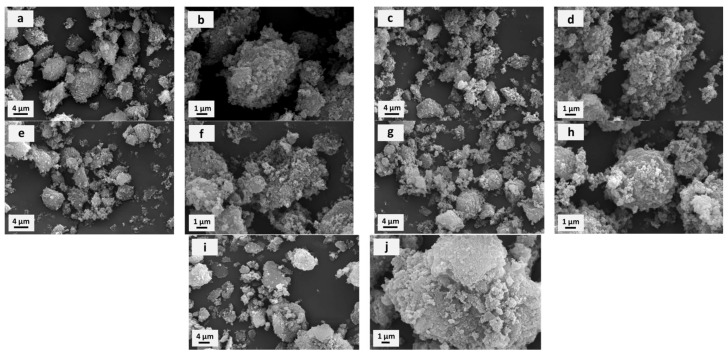
Scanning electron microscopy (SEM) images of initial rCB (**a**,**b**) and modified samples: rCB_SA_3h (**c**,**d**), rCB_SA_24h (**e**,**f**), rCB_BDS_20h (**g**,**h**), and G/rCB_SA_3h (**i**,**j**).

The morphology of the initial rCB and the modified samples, obtained through SEM and TEM micrographs, is depicted in Figure 2 and Figure S1. As shown in Figure 2a, rCB consists of large, irregularly shaped particles with rough surfaces. At higher magnification in Figure 2b, these fragments appear as clusters or aggregates of smaller particles bound together, forming a rough and porous texture. The smaller particles within the aggregates exhibit a roughly spherical to irregular shape. Additionally, they are not tightly arranged, generating void spaces or gaps between them, which may contribute to the sample’s porosity, as also indicated in Table 1. The SEM images obtained for the SA- or BDS-modified rCB (Figure 2c–f) did not differ significantly from those of the unmodified sample (Figure 2a,b), suggesting that the activating agents used slightly affected the rCB morphology. Only some minor changes in the aggregates’ size and shape were observed for the rCB_SA_3h (the aggregates appear smaller and resemble a coral reef), which is probably related to the removal of mineral matter from rCB (see also ash content in Table 2). The morphology of the G/rCB_SA_3h composite is somewhat different from the other samples. The aggregates are larger, and the primary particles are more irregular in shape and more tightly packed. However, voids and gaps are still visible within the aggregates’ structure, contributing to the sample’s porosity (see also Table 1).

The morphology of the selected rCB samples was further confirmed through TEM micrographs, as illustrated in Figure S1. Both the initial rCB and the selected sulfonated samples predominantly consist of spherical particles with a broad and varied size distribution. These particles exhibit a pronounced tendency to form agglomerates, particularly when the sulfonation time was extended (rCB_SA_24h) and when BDS was used as the sulfonating agent (rCB_BDS_20h).

**Figure 3:**
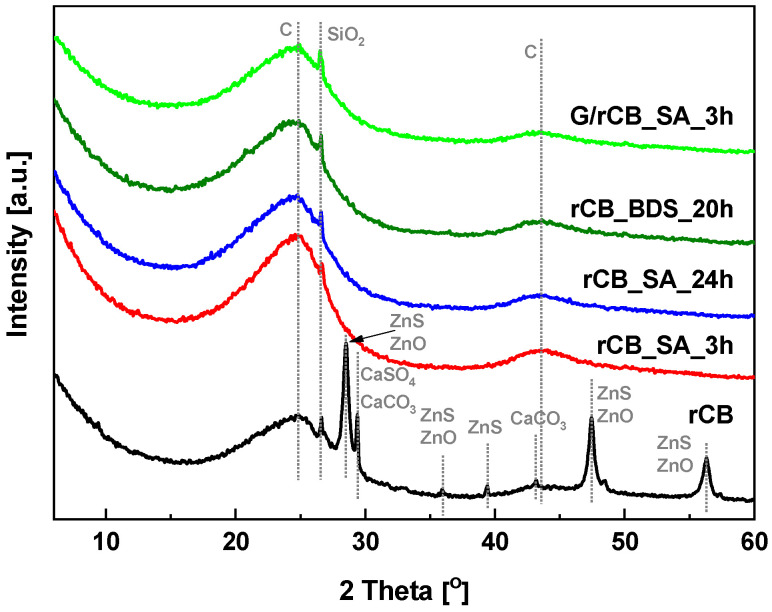
PXRD patterns of the initial rCB and modified samples.

The elemental composition of the initial rCB and modified samples is shown in Table 2. The carbon and hydrogen contents in the initial rCB and the catalysts treated with SA or BDS are very similar, measuring 68.5–72.4% and around 1%, respectively. The lower carbon content in the rCB samples, compared to the typical composition of carbon blacks, is attributed to a substantial amount of ash present in the samples, ranging from 13.6% to 25.5%. The presence of minerals on the rCB surface was also confirmed by PXRD results (see Figure 3). Notably, the initial rCB sample exhibits a substantial amount of sulfur, about 2.2%, resulting from the sulfur in the tire feedstock used during the vulcanization process. This sulfur value is similar to that reported by other authors [49,50]. The formation of acidic groups on the surface of carbon materials leads to a higher oxygen content, about 4.2–6%, compared to the initial rCB sample. The increase in oxygen content, apart from that contained in sulfonic groups, is likely due to oxidation/dehydrogenation processes occurring alongside sulfonation with sulfuric acid [51]. The highest oxygen content, around 12%, and hydrogen content, about 1.7%, are observed in the composites obtained by the partial carbonization of glucose on the surface of the carbon material. The formation of weak acidic centers, such as COOH and OH, due to the incomplete carbonization of the organic precursor has been previously reported in the literature and could account for this observation [48].

The results of the total acidity (A_tot_) and density of acidic centers of the materials are presented in Table 2. As observed, the initial rCB exhibited a low A_tot_ of 0.1 mmol H^+^/g, most likely due to the presence of small amounts of oxygen in the sample (Table 2). The functionalization significantly increased the acidity of rCB by introducing -SO_3_H and oxygen groups on the sample surface (for details see further analyses of XPS and FT-IR). The observed effect was dependent on the type of modifying agent. The least efficient approach for inducing acidity was using BDS, which resulted in a sample with an A_tot_ of 0.5 mmol H^+^/g. In contrast, the rCB_SA samples, obtained by pretreating the pyrolytic rCB exclusively with sulfuric acid, achieved an A_tot_ of approximately 1.0 mmol H^+^/g. The acidity of rCB_BDS_20h likely stemmed from the anchoring of -PhSO_3_H on the rCB surface despite the modified sample containing less S than the initial rCB (see Table 2). This effect results from two opposing processes occurring during the rCB modifications: (i) the introduction of new S-containing groups into the sample matrix, and (ii) the removal of S originally present in the sample in the form of mineral matter (see ash contents in Table 2 and PXRD analysis in Figure 3). Additionally, some authors have claimed that the formation of internal salts, which results in the neutralization of -SO_3_H sites, can be responsible for the lower acidity of the sample modified with BDS [52]. Interestingly, the acidities of rCB functionalized with SA for 3 and 24 h did not differ significantly (1.0 and 0.9 mmol H^+^/g, respectively), favoring the approach using a shorter modification time. The highest total acidity was noted for the G/rCB composite modified with sulfuric acid (1.9 mmol H^+^/g), attributed not only to sulfonic groups but also to oxygen functionalities (see increased S and O contents for G/rCB_SA_3h compared to rCB in Table 2).

Powder XRD analysis of all the rCB samples is depicted in Figure 3. As shown, the initial rCB material exhibits an amorphous structure evidenced by a broad signal at 2θ with a maximum at around 24° (002 crystal plane) and a weak reflection at 2θ around 43° (100 crystal plane), typical for carbon materials [53]. The PXRD pattern of the rCB material also reveals the presence of impurities, mainly zinc in the forms of ZnS and ZnO, characterized by peaks at 2θ around 28.5°, 36°, 48°, and 56° [54,55,56,57]. Moreover, CaCO_3_ and CaSO_4_ compounds are indicted by a signal at 2θ equal to 29°, while SiO_2_ is identified by a small reflection at 2θ equal to 26.6° [54,55]. Additionally, the reflection at ca. 2θ around 43° observed in the initial rCB sample confirms the presence of CaCO_3_. The presence of such compounds results from tire compositions [58,59] and their persistence as residue after the pyrolysis process [56]. This also aligns with the ash content data presented in Table 2. 

Modification of rCB material with concentrated sulfuric acid (SA) and BDS, as well as glucose carbonization followed by SA treatment, resulted in the vanishing of most reflections attributed to inorganic impurities. This suggests that these modifications effectively removed a significant portion of the mineral matter from the rCB material. This finding is consistent with the results in Table 2, which show decreased ash contents in the acid-functionalized rCB samples. Besides the broad bands indicating the amorphous structure of the carbon materials, only weak silica reflections were present in the PXRD patterns of the modified samples. 

**Figure 4:**
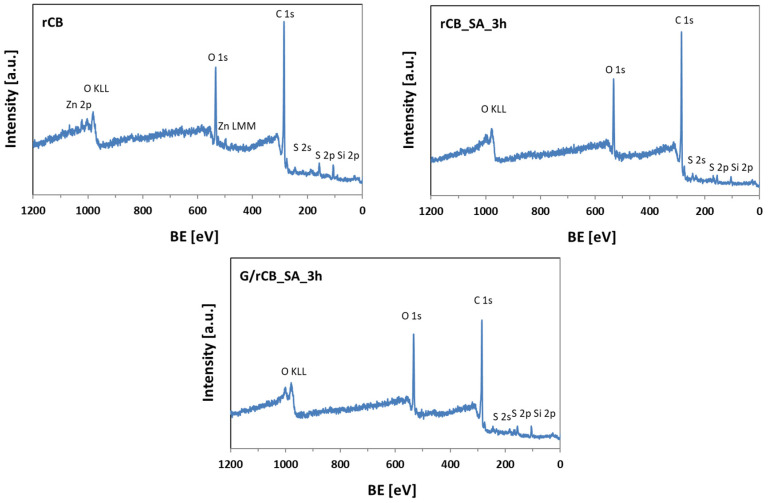
The XPS survey spectra of indicated samples.

The surface chemical structure of selected samples was analyzed using X-ray photoelectron spectroscopy (XPS). The survey spectra of the initial rCB and a selected modified material in Figure 4 displayed well-resolved C 1s and O 1s peaks, along with lower-intensity peaks corresponding to Si 2p, Zn 2p, and S 2p for rCB, as well as to Si 2p and S 2p in functionalized carbons. According to the data presented in Table 3, rCB contained sulfur in its structure, which aligns with the data from elemental analysis (EA) shown in Table 2. Unlike EA, the XPS method revealed a very high oxygen content in the sample. This discrepancy is attributed to XPS’s ability to detect oxygen in various chemical forms on the surface, such as salts or oxides, whereas in EA, oxygen must be fully released and converted to a gaseous form for detection. Importantly, the functionalized samples exhibited an increased sulfur content compared to rCB, suggesting the successful introduction of new functionalities onto the carbon surface during the modifications.

**Figure 5:**
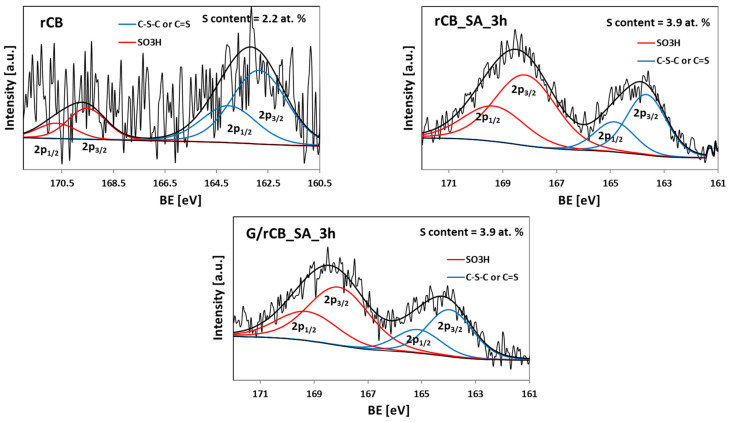
High-resolution XPS S2p spectra of the selected rCB samples.

A high-resolution XPS S2p spectra of rCB and selected modified carbons (Figure 5) showed that sulfur was present in the samples in different oxidation states. All the spectra exhibited signals around 164 and 168 eV (observed as a doublet), which can be attributed to the reduced and oxidized forms of S, respectively [23,60]. The contribution of these signals, however, varied between samples. In the rCB, sulfur was predominantly in the reduced form (peaks at 163.8 and 165.0 eV, assigned to -C-S-C- sulfide bridges, dominated) [61]. Conversely, in the functionalized carbons, sulfur was primarily in its oxidized state, with a high-energy doublet at 167.9 and 169.1 eV attributed to the -C-SO_x_-C- groups (where x = 2 or 3) being dominant [23,62]. This indicates the successful introduction of -SO_3_H groups during the functionalization (Figure 5). The relative concentrations of different S-containing surface groups are summarized in Table 4.

XPS measurements revealed that different sulfur-containing groups were predominant in the initial rCB material (-C-S-C-) compared to the modified rCB samples, where -C-SO_x_-C- groups were more prominent. Given that sulfur has two electron pairs [63], modifying carbon materials with sulfuric acid can influence the electronic structure of the system. Although sulfur’s electronegativity is similar to carbon’s (2.58 vs. 2.55), its larger atomic radius (102 pm compared to carbon’s 75 pm) leads to an increase in defects when sulfur atoms are introduced into carbon materials. Altering the electronic structure of materials with potential catalytic applications has been shown to significantly impact catalytic performance, as highlighted in the literature [64].

**Figure 6:**
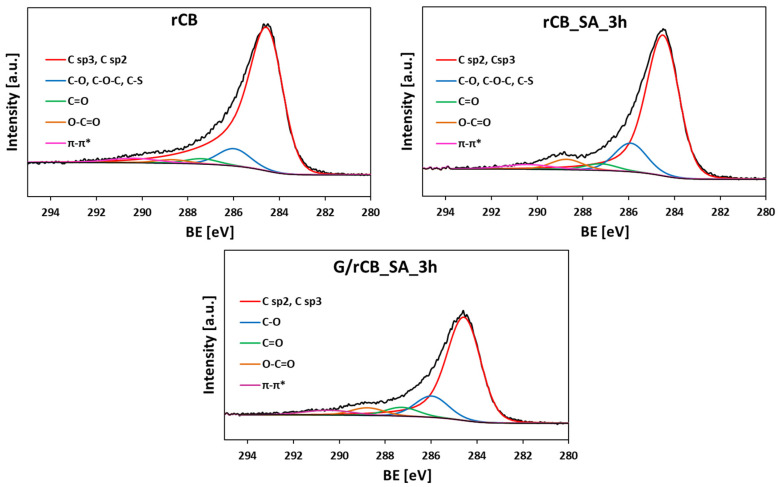
High-resolution XPS C1s spectra of the selected rCB samples.

Figure 6 shows high-resolution XPS C 1s spectra of the initial rCB and selected modified samples. All these spectra can be deconvoluted into peaks centered at ~284.5 eV, 285.9 eV, 287.3 eV, 289.6 eV, and 290.6 eV (±0.1–0.2 eV in each case), typically assigned to (i) sp^2^ and sp^3^ hybridized carbon, (ii) C-O in hydroxyl/phenolic and ether groups and C-S, (iii) C=O in carbonyls, (iv) O-C=O in carboxylic acids, esters, and inorganic carbonates, and (v) π–π* transitions, respectively [23,65,66]. The relative abundance of different carbon species derived from the deconvoluted spectra is listed in Table 5. The results show that graphitic carbon predominates in all investigated samples, constituting more than 66%. The samples also contain a relatively high amount of moieties in which carbon is singly bound to an oxygen atom (C-O).

**Figure 7:**
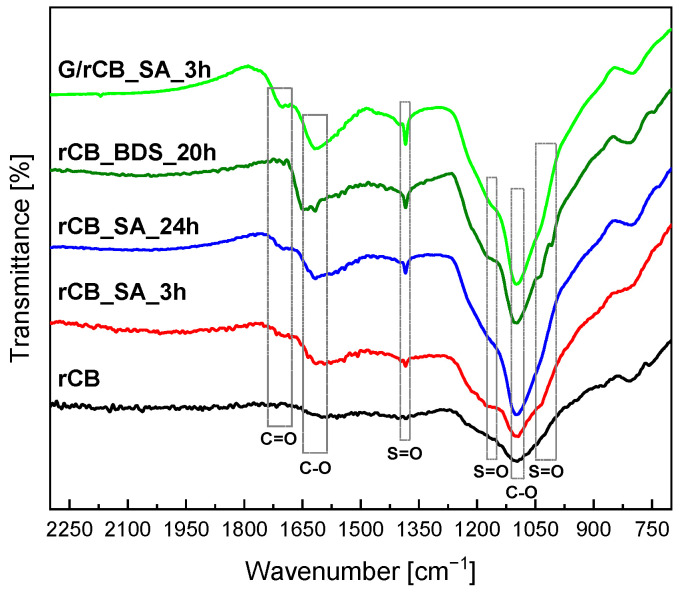
FT−IR spectra of initial and modified rCB samples before the reaction cycle (initial catalysts).

Figure 7 presents the FT-IR spectra of the initial rCB and the modified samples. As observed, rCB exhibits a broad band with a maximum of about 1100 cm^−1^, which is attributed to the presence of carboxyl groups [8]. This band overlaps with the band characteristic of the O=S=O group. Analysis of FT-IR spectra of acid-modified materials (Figure 7) shows that treatment with sulfuric acid and BDS generates new bands at about 1040 and 1380 cm^−1^, as well as a weak band at 1183 cm^−1^ [67,68]. According to Zhao et al. [68], these bands indicate the formation of SO_2_ (O=S=O) asymmetric and symmetric stretching vibrations. Notably, the bands at about 1380 cm^−1^ (stretching vibration of SO_3_) and 1040 cm^−1^ (stretching vibration of SO_3_H) are more pronounced for rCB modified with glucose followed by sulfonation compared to those directly sulfonated. Additionally, according to the literature [69], bands at approximately 1710 cm^−1^ and 1650 cm^−1^ indicate the presence of carboxyl and carbonyl bonds in acidic surface groups. This finding is consistent with the acidity measurement results (Table 2). 

**Figure 8:**
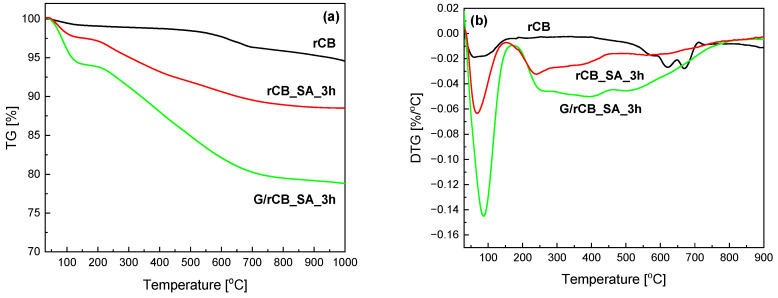
TG (**a**) and DTG (**b**) analysis of rCB and selected modified samples (nitrogen atmosphere).

Thermogravimetric analysis performed under an inert gas flow further reflects the effectiveness of sample functionalization. Figure 8 shows the results of the TG and DTG analyses carried out for the initial recovered carbon black (rCB) and selected samples after functionalization. As observed in Figure 8a, rCB shows a slight weight loss in two temperature ranges seen in the DTG curves (Figure 8b). A small initial effect (~1% weight loss) was recorded in the temperature range of 25–100 °C due to the presence of physically adsorbed water. The second weight loss (~3%) in the range of 530–700 °C is likely related to the presence of inorganic additives identified as ash contents (refer to Table 2 and PXRD patterns in Figure 3) and further carbonization of a rubber-derived carbonaceous solid [70]. This effect is not pronounced in the modified materials, confirming the partial removal of mineral matter and impurities from the pure rCB material during treatment with sulfuric acid. In contrast, the modified materials exhibited significantly lower thermal stability compared to the parent sample, indicating efficient functionalization with new functional groups. Notably, a higher weight loss of approximately 11% was observed for the sample treated with sulfuric acid (rCB_SA_3h) and around 20% for the composite of rCB with glucose (G/rCB_SA_3h). This observation is also consistent with the S and O contents reported in Table 2. The weight loss for the modified samples is observed in two ranges: around 100 °C (for rCB_SA_3h—weight loss of 2% and G/rCB_SA_3h—6%) and broad signals in the temperature ranges of approximately 200–750 °C, with maxima at around 250, 400, and 510 °C (Figure 8b). The significantly higher mass loss at low temperatures, resulting from water removal, may be attributed to the more hydrophilic nature of the samples after modification due to the high density of hydrophilic functional groups bound to carbon. According to the literature data, the weight loss in the second-stage temperature range may be due to the decomposition of sulfonate groups present on the surface of the samples, as well as the presence of oxygen functional groups [71,72]. A similar effect was observed in our earlier work [23]. The presence of such groups results from the oxidizing properties of concentrated sulfuric acid [23]. In the case of the G/rCB_SA_3h composite, the oxygen-rich sample structure is also due to the method of preparation (see also Materials and Methods); i.e., low-temperature treatment of glucose typically produces oxygen-abundant (hydro)char [73]. The presence of these effects confirms the effective introduction of functional groups on the surface of the studied rCBs. 

### 2.2. Catalytic Activity in Glycerol Acetalization Reaction with Acetone

Our previous research focused on synthesizing solketal as a potentially important fuel component using modified zeolite mesoporous matrices as catalysts due to their broad range of applications and economical large-scale production [40,41]. In this study, we explored the use of recovered carbon black (rCB) material as a catalyst for the acetalization reaction of glycerol. This approach is particularly promising for recycling waste materials. Notably, the use of carbon materials in the production of solketal has not been extensively studied [19,33,74,75,76] despite their demonstrated potential in other catalytic processes [23,77,78,79,80,81]. Therefore, in the present study, we used recovered carbon black materials, a by-product of waste tire pyrolysis, which were additionally functionalized with acidic groups as catalysts in the acetalization reaction of glycerol with acetone. The catalyst’s activity was tested using a sulfuric acid-modified sample across a temperature range of 30–70 °C, with reaction times of 0.5–1 h, under atmospheric pressure, and an acetone-to-glycerol molar ratio of 1:1 (Figure S2). The optimal conditions for producing solketal were found to be a temperature of 40 °C, with maximum glycerol conversion achieved after 0.5 h. These conditions were therefore applied in subsequent experiments. Further increases in glycerol conversion with rising temperatures were minimal, suggesting that the conditions proposed in our research are economically attractive. 

**Figure 9:**
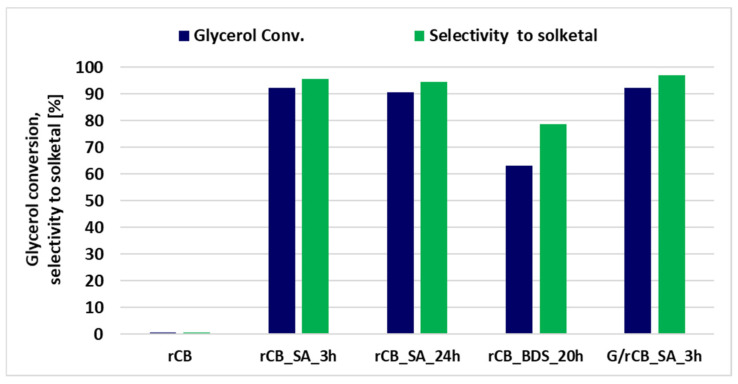
The influence of different methods of acidic group formation in rCB materials on glycerol conversion and selectivity to solketal, evaluated at 40 °C for 0.5 h.

Given that the unmodified rCB material showed practically no catalytic activity in the reaction tested (Figure 9), its performance was equal to that of the blank test, which did not use a catalyst. We presume this lack of activity is due to the absence of acid groups involved in the acetalization reaction. To address this, we attempted to functionalize the rCB surface to generate catalytically active sites. The modifications aimed at introducing sulfonic groups were achieved by treating the rCB with sulfuric acid (SA) or 4-benzenediazonium sulfonate (BDS). Additionally, the glucose/rCB (G/rCB) composite was prepared by first modifying the rCB surface with glucose, followed by partial carbonization, and then treating it with sulfuric acid (SA). The goal of this modification was to stabilize the material after partial carbonization and subsequent sulfonation with SA. This approach is similar to methods reported in previous studies [82]. 

Among the two modifiers used to introduce sulfonic groups onto the carbon material, treatment with sulfuric acid yielded significantly better results when compared to BDS alteration (Figure 9). Glycerol conversion on rCB treated with sulfuric acid (SA) for 3 h exceeded 92%, with a selectivity of 96% for the desired product, solketal. On the other hand, the introduction of glucose onto the surface of the rCB material with the following partial carbonization and sulfonation did not affect glycerol conversion and only slightly improved selectivity to solketal. In contrast, the sample modified with 4-benzenediazonium sulfonate (BDS) achieved only 60% glycerol conversion and 78% selectivity to solketal. Along with solketal, a small amount of the isomeric compound 1,3-dioxan-5-ol was also produced. The reaction between glycerol and acetone is a reversible process, resulting in the formation of 2,2-dimethyl-1,3-dioxolane-4-methanol (solketal), isomer, and water (as illustrated in Scheme S1).

Although solketal is a kinetically stable product, the formation of the thermodynamically stable isomer (2,2-dimethyl-1,3-dioxan-5-ol) is also observed. The advantage of the applied rCB materials, especially modified by means of SA treatment, is their ability to achieve a high glycerol conversion and excellent selectivity for solketal after a relatively short sulfonation process of just three hours. To investigate the effect of sulfonation time on the catalytic activity of the rCB material, a series of experiments with varying sulfonation durations were conducted. The study revealed that a three-hour sulfonation period is optimal, and extending the treatment time further is unnecessary, as supported by recommendations in the literature [23,70].

**Figure 10:**
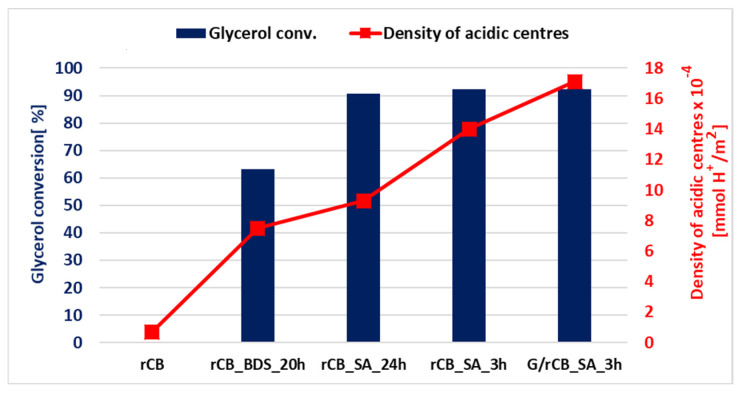
The dependence of the density of acidic centers on glycerol conversion in the acetalization reaction. The density of acidic centers was calculated as acidity [mmol H^+^/g] divided by S_BET_ [m^2^/g].

The modifications with sulfuric acid (SA) and 4-benzenediazonium sulfonate (BDS) lead to the formation of active sites with acidic functions, as shown in Table 2. The increase in glycerol conversion correlates with a higher density of acid sites (Figure 10, Table 2). The importance of Brønsted acidic functions in the glycerol acetalization reaction with acetone has been highlighted in numerous studies [42,43,44,45]. In the proposed mechanism, the activation of the acetone carbonyl group by the hydrogen of the SO_3_H group occurs, followed by a nucleophilic attack by the alcohol group of glycerol, leading to the formation of a bond between the carbonyl oxygen atom and the β-carbon of glycerol. This process results in dehydration and the formation of the five-membered ring of solketal (Scheme S2).

**Figure 11:**
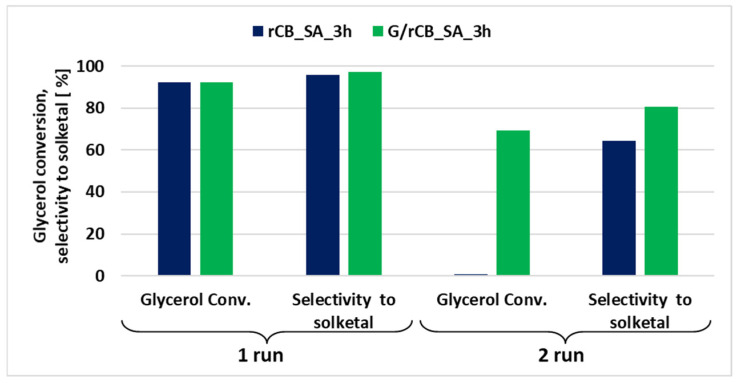
The catalyst activity in 1st and 2nd reaction cycle. Acetalization reaction conditions of glycerol with acetone: acetone-to-glycerol molar ratio of 1:1, temperature 40 °C, time 0.5 h.

Not only activity but also the stability of catalysts is a crucial factor for their feasibility in industrial-scale applications. To assess their potential for reuse, the acetalization reaction of glycerol with acetone was tested using both a sulfonated catalyst and rCB impregnated with glucose followed by the sulfonation process. The activity of these catalysts was evaluated over two reaction cycles (Figure 11). All catalysts exhibited satisfactory initial activity, with glycerol conversion exceeding 92% and selectivity for solketal reaching 97%. However, the rCB_SA_3h catalyst, which was solely functionalized through sulfonation, showed a dramatic decrease in activity to below 1% in the second cycle. In contrast, only the catalyst obtained through the dual functionalization process—impregnation with glucose followed by partial carbonization and sulfonation—remained active in the second cycle. For this catalyst, glycerol conversion exceeded 69%, and the selectivity to solketal was around 80%.

To investigate the differences in activity among the reused samples, we conducted FT-IR studies on the spent catalysts. The FT-IR spectra of the spent rCB catalysts (Figure S3) still show bands characteristic of sulfur-containing groups (1380, 1040 cm^−1^), as well as a less intensive band from the stretching vibrations of carboxyl groups (C=O) around 1700 cm^−1^ [82]. This latter band is more pronounced for rCB impregnated with glucose followed by sulfonation, which correlates with the relatively high activity of this catalyst in the subsequent run (Figure 11). On the other hand, the FT-IR spectrum of spent rCB directly modified with sulfuric acid for 3 h indicates the presence of mainly sulfuric acid groups, whereas the carboxylic groups are hardly visible. Despite the presence of some acidic groups in spent rCB_SA_3h, this catalyst was completely deactivated after the first use. Therefore, it appears reasonable to conclude that sulfonic acid groups are not the sole active sites in glycerol acetalization. The carboxylic groups, whose presence on spent G/rCB sample was inferred from the higher carbon and oxygen content confirmed by elemental analysis (Table 2) and FT-IR spectra (Figure S3), also play a significant role in this process. The possible activity of -COOH groups in solketal formation was also indicated by other authors [33].

Looking for some further explanation of the observed catalytic results, the spent catalysts were also characterized using XPS spectroscopy (please refer to Tables S1–S3 and Figures S4–S6). After the reaction, in the case of the glucose-modified sample, the amount of graphitic carbon decreased with the simultaneous increase in content of carbon single and double bonded to oxygen. Sample rCB_SA_3h after the reaction shows an increase only in carbon double-bonded to oxygen at the expense of graphitic carbon (Figure S5 and Table S2). Furthermore, significant changes in sulfur content were observed in all spent catalysts, regardless of the preparation method (Figure S6 and Table S3). Notably, although sulfur content decreased more dramatically in the rCB/glucose composite, this catalyst maintained relatively high activity even in subsequent reaction cycles. This suggests that several factors contribute to catalyst deactivation. However, the reduction in sulfur content appears to be only one possible explanation for the decreased activity of the spent catalysts. The results clearly indicate that sulfonic groups are not the sole active acid sites in the studied catalysts. This is supported by the acidity measurements, which show that the rCB/glucose composite has significantly higher total acidity (A_tot_) values despite having similar sulfur content to the rCB_SA sample. This further supports that carboxylic groups are also important in glycerol acetalization. Their presence likely contributes to the relatively high activity of the rCB/glucose composite in the second reaction cycle, despite the observed decrease in sulfur content. The presence of hydrophilic –COOH groups has been discussed in the literature, highlighting their potential role in enhancing the catalyst’s activity by creating a strong affinity between the hydrophilic parts of the reactants and the catalyst [75].

Different activated carbons were applied as catalysts for synthesis of solketal. Table 6 presents a comparison of the catalytic performance achieved in glycerol acetalization over rCB divided from end-of-life tires and other carbon-type catalysts. As observed, the glycerol conversion obtained using carbons catalysts described in the literature was consistently high and comparable (in the range of 80–90%) across all cases. However, the conditions required to achieve these satisfactory results varied among the studies. For instance, Rodrigues et al. [83] found that activated carbon (AC) prepared by chemical activation of olive stone wastes could give more than 60% glycerol conversion within 6 h. However, a large excess of acetone (glycerol to acetone molar ratio of 1:4) and greater catalyst content (Table 6, line 2) were necessary to obtain results comparable to those presented in other studies. Other types of acid carbons (CAs), obtained by hydrothermal carbonization of a mixture of glycerol and sulfuric acid, were effective using a glycerol-to-acetone molar ratio of 1:3, achieving 84% glycerol conversion within 2 h [33]. On the other hand, the carbon produced from bio-oil and sulfuric acid (BS) showed excellent glycerol conversion using a glycerol-to-propanone molar ratio of 1:10, within 2 h [19]. In contrast, Nandan et al. [75], who investigated thermally (SCS1) and hydrothermally (HSCS1) activated sulphonated carbon–silica meso composite materials, found that the higher activity of HSCS1 was related to the higher acidity of these materials. All the carbon materials presented were functionalized or synthesized in the presence of sulfuric acid, which was a source of Brønsted acid sites. In some cases, the synthesis of these systems was sophisticated and required additional preparation steps. In view of the above, the catalytic performance of functionalized rCB, representing industrial waste, was very promising, as the samples achieved significant glycerol conversion (more than 90%) in a short time using an economically viable glycerol-to-acetone molar ratio of 1:1. This clearly demonstrates that problematic end-of-life tires can be successfully valorized into efficient carbon-based catalysts with promising activities in solketal formation, contributing to the recycling of troublesome rubber waste.

## 3. Materials and Methods

Recovered carbon black (rCB) in a pelletized form was supplied by Contec S.A. Poland and produced from used tires in a continuous waste tire pyrolysis process at 510 °C, using the so-called Molten technology [84]. Concentrated sulfuric acid (p.a., 96 wt.%) was purchased from Carlo Erba. Sulfanilic acid (p.a., 99 wt.%) was obtained from Chemat, sodium nitrite from Chempur, and hydrochloric acid (p.a., 35–38 wt.%) from Stanlab. The reagents used in the catalytic experiments—glycerol (p.a., 99.5 wt.%), acetone (p.a., 99 wt.%), and methanol (p.a., 99.8 wt.%)—were all purchased from Stanlab.

### 3.1. Functionalization of rCB

rCB was functionalized to endow the sample surface with acidic features. This was performed by modifying rCB with concentrated sulfuric acid or generated in situ with diazonium salt. Details of these functionalizations are presented below.

The process using sulfuric acid (denoted as ‘SA’) as a source of -SO_3_H groups was performed under an Ar flow, with continuous stirring in a three-neck round-bottom flask equipped with a condenser. In each case, 3.5 g of the sample and 90 mL of H_2_SO_4_ were mixed and heated to 140 °C. The reaction was carried out for various durations: 3 and 24 h. After the reaction, the mixture was cooled, diluted with distilled water, and filtered. The collected sample was then thoroughly washed with hot distilled water until the filtrate pH was neutral. Finally, the material was dried at 105 °C overnight. The samples were labeled according to the scheme: rCB_SA_3h, where SA stands for concentrated sulfuric acid, and 3h denotes the sulfonation process duration.

The procedure for introducing sulfonic groups on the surface of carbon material from in situ formed 4-benzenediazonium sulfonate (donated as ‘BDS’) was carried out using the same apparatus employed for the modification with SA. The sulfonation reaction took place under a reflux condenser at room temperature with vigorous stirring. For the reaction, 1.75 g of the test matrix was combined with 90 mL of distilled water, and the mixture was stirred vigorously for 30 min. Then, 2.5 g of sulfanilic acid was added and stirred until dissolved. Next, 1 g of sodium nitrite was added, and the stirring was continued for another 30 min. Consequently, 4-benzenediazonium sulfonate was formed in situ in the reaction between 4-aminobenzenesulfonic acid and sodium nitrite in water. Subsequently, 17.5 mL of concentrated hydrochloric acid was slowly added over the course of one hour. After 20 h, the resulting solution was diluted with distilled water and then filtered. The modified carbon material was washed with distilled, hot water. The washing procedure was repeated until the filtrate pH was neutral, after which the obtained material was washed twice with 50 mL each of methanol, DMF, and acetone to remove organic residues. Finally, the washed sample of modified carbon material was dried at 105 °C for 24 h. The sample was labeled as rCB_BDS_20h. Details of the modification procedures used can be found elsewhere [79].

### 3.2. Preparation of rCB Composite

The rCB material was modified using glucose to produce composite with enhanced acidity and stability. The composite was performed via impregnation method with glucose followed by carbonization of the deposited sugar.

This method was based on the procedure proposed by Mo et al. [82]. Briefly, the pre-dried rCB material (~1 g) was impregnated with a mixture of 0.5 g of glucose, 10 mL of deionized water, and 0.2 g of H_2_SO_4_. After drying at 105 °C, the sample was carbonized at 180 °C for 20 h under Ar flow. The prepared composite was subsequently sulfonated with SA for 3 h using the standard conditions described above. The obtained catalyst was labeled G/rCB_SA_3h.

### 3.3. Physicochemical Characterization of the Catalysts

Elemental analysis was performed using a FLASH 2000 instrument from Thermo Scientific, USA. The elemental content (C, H, N, S, and O) was determined using a standard curve. The ash contents were determined as the difference (100%-CHNSO%).

PXRD measurements were conducted using powder X-ray diffraction on a BRUKER D8 ADVANCE diffractometer equipped with a Cu lamp (Cu Kα1) emitting radiation with a wavelength of λ = 0.15406 nm. The analysis was conducted in the wide-angle (6–60°) range.

The FT-IR spectra were recorded using a Bruker Tensor 27 spectrophotometer. Measurements were performed using the transmission technique in the wavenumber range of 4000–400 cm^−1^ with a resolution of 1 cm^−1^. The sample was mixed with 200 mg of KBr and formed into pellets using a press (150 MPa).

The total acidity of the materials was measured using a back titration method. Approximately 100 mg of the sample, which was first activated at 105 °C for 24 h, was mixed with 50 mL of a 0.01 M NaOH solution and allowed to stand at room temperature for 24 h. Afterwards, the suspensions were filtered, and the filtrate was titrated with a 0.01 M HCl solution. A blank test (without a sample) was also performed, and the obtained value was used for the calculations.

The Brunauer–Emmet–Teller (BET) surface areas (S_BET_) were determined by N_2_ adsorption at −196 °C using a Micromeritics ASAP 2010 sorptometer. Total pore volume (V_BJH_) and average pore diameter (D) were determined by the Barrett–Joyner–Halenda (BJH) method to the desorption branch of the isotherm. The total pore volume (V_tot_) was measured from the amount of N_2_ adsorbed at p/p_0_ = 0.99. Prior to the measurements of adsorption–desorption isotherms, the samples were outgassed at 90 °C for 8 h. The microporous surface area (S_micro_) and volume (V_micro_) were determined using the t-plot method.

X-ray photoelectron spectroscopy (XPS) measurements were conducted using an Ultra-High-Vacuum (UHV) System (Specs, Berlin, Germany). The examined materials were irradiated with a monochromatic Al Kα radiation (1486.6 eV). The operating pressure in the chamber was close to 2 × 10^−9^ mbar. Binding energies (BEs) were calibrated against the C1s peak set at 284.5 eV. Spectroscopic data were processed through CasaXPS software (version 2.3.22PR1.0) developed by Casa Soft-ware Ltd., Teignmouth, UK, employing a peak-fitting algorithm with a non-linear Shirley-type background correction.

Material morphology was examined using scanning electron microscopy (SEM) with a Hitachi SU3500 instrument. Transmission electron microscopy (TEM) images were acquired using a JEOL 2000 microscope with an accelerating voltage of 80 kV. The materials studied were deposited on copper grids coated with a carbon film.

Thermogravimetric analysis was performed using SetSYS 1200 device (Setaram). The measurements were performed in a nitrogen atmosphere by heating about 1g of the sample to a temperature of 1000 °C with an increase of 10 °C/min.

### 3.4. Catalytic Tests

All tested carbon materials, both initial and modified, were evaluated as heterogeneous catalysts in the acetalization reaction of glycerol with acetone. Prior to catalytic testing, the materials underwent a 24 h activation process at 105 °C. In a typical experiment, the catalytic activity was assessed in a system comprising 1 g of glycerol, 0.8 mL of acetone, and 10 mg of catalyst. The acetalization reaction was conducted in closed glass vials on a magnetic stirrer. The reaction was performed at temperatures of 30 °C, 40 °C, and 70 °C, for approximately 0.5–1 h, to optimize the method. After the reaction time elapsed, the samples were cooled to room temperature, and 1 mL of dried methanol was added. The solid material was separated using a syringe filter, and the filtrate was analyzed using a Varian CP-3800 gas chromatograph equipped with a flame ionization detector (FID) and a VF-5ms capillary column (30 m length, 250 µm internal diameter). Product identification was achieved by comparing the retention times of the obtained peaks with those of standard substances. The reaction mixture contained unreacted glycerol, as well as acetone, methanol, solketal, 2,2-dimethyl-1,3-dioxolane-4-methanol and its isomer, 2,2-dimethyl-1,3-dioxolane-5-ol. The conversion of glycerol (denoted as Glycerol conv.), selectivity to products: solketal (denoted as Selectivity to solketal), and isomer were calculated according to the equations presented in the referenced paper [41]. The reuse of catalysts involved crucial steps such as washing and drying. Following the first reaction cycle, the catalyst was washed three times with 25 mL of methanol. It was then dried at 105 °C for 24 h. After cooling, the catalyst was ready for reuse in the second reaction cycle.

## 4. Conclusions

Acid-modified pyrolysis-recovered carbon black materials derived from post-consumer tires were prepared through a sulfonation process using concentrated sulfuric acid or BDS. The sulfonation procedure significantly influenced the properties of the acid-carbon catalysts. All sulfonated carbons exhibited high activity in the synthesis of solketal at 40 °C in a batch reactor. The functionalized rCB demonstrated excellent performance in glycerol acetalization, achieving a glycerol conversion rate of approximately 90% within just 30 min, with solketal selectivity exceeding 95%. The optimal results were obtained with samples treated with concentrated sulfuric acid for 3 h, achieving a solketal yield of around 90%.

Variations in glycerol acetalization performance were attributed to differences in structural parameters (e.g., surface area) and the presence of acidic functional groups (namely sulfonic and carbonyl). It was found that sulfonic groups are not the only active centers in glycerol acetalization and carbonyl groups also play a significant role. The BDS-modified rCB showed generally lower activity, likely due to partial pore blocking and limited access of reactants to the catalytic sites.

Reusability tests indicated that additional modification with glucose stabilized the acid-carbon catalytic system, enhancing the durability of the sulfonated pyrolytic recovered carbon black in a subsequent catalytic run.

The obtained results indicate a significant impact of the methods used for the acidic modification of carbon materials. The introduction of glucose into the modified materials, followed by incomplete carbonization and sulfonation, appears to be the most promising approach. Therefore, the subsequent research will focus on various methods of rCB acidification, considering the introduction of sulfuric acid at different stages of waste tire carbonization, as well as varying the time and amount of acidifying agents used. Particularly, the improvement in the stability of modified samples, as well as the role of ash presence on catalytic activity and the effect of its removal, will be the subject of future research.

## Figures and Tables

**Table 1:** Physicochemical characterization of the initial rCB and modified samples.

Sample	Sulfonation Time [hour]	Source of -SO_3_H Group	S_BET_^a^ [m^2^/g]	S_micro_^b^ [m^2^/g]	S_meso_^c^ [m^2^/g]	V_tot_^d^ [cm^3^/g]	V_micro_^e^ [cm^3^/g ×10^−4^]	D ^f^ [nm]
rCB	-	-	75	3	72	0.22	0.35	28.7
rCB_SA_3h	3	H_2_SO_4_	68	13	55	0.19	54.61	31.1
rCB_SA_24h	24	H_2_SO_4_	91	17	74	0.26	71.47	26.0
rCB_BDS_20h	20	C_6_H_7_NO_3_S	60	3	57	0.20	3.98	28.6
G/rCB_SA_3h	3	H_2_SO_4_	111	44	67	0.12	196.74	13.6

^a^ S_BET_—BET specific surface area, ^b^ S_micro_—micropore area from the t-plot method, ^c^ S_meso_—external surface area (mesopore area) from the t-plot method, ^d^ V_tot_—single point total pore volume at p/p_0_ = 0.99, ^e^ V_micro_—micropore volume from the t-plot method, ^f^ D—BJH desorption average pore diameter.

**Table 2:** The elemental composition, ash content, and total acidity obtained for the initial rCB and modified samples.

Sample	C [wt.%]	H [wt.%]	N [wt.%]	S [wt.%]	O ^a^ [wt.%]	Ash ^b^ [wt.%]	Total Acidity ^c^ [mmol H^+^/g]	Density of Acid Centers ^d^ [mmol H^+^/m^2^ ×10^−4^]
rCB	68.5	0.8	0.1	2.2	2.9	25.5	0.1	0.7
rCB_SA_3h	77.0	0.9	0.1	2.3	6.1	13.6	1.0	14.0
rCB_SA_24h	69.7	0.7	0.1	1.7	6.1	21.7	0.9	9.3
rCB_BDS_20h	72.4	1.0	0.3	1.5	4.2	20.6	0.5	7.5
G/rCB_SA_3h	68.5	1.7	0.1	2.3	11.9	15.5	1.9	17.1

^a^ determined using a FLASH 2000 Thermo Scientific instrument; ^b^ calculated by the difference (100%-CHNSO%), ^c^ the total acidity of the materials was measured using a back titration method using Tashiro indicator, ^d^ density of acidic centers calculted as follows: total acidity [mmol H^+^/g] divided by S_BET_ [m^2^/g].

**Table 3:** Elemental composition of selected rCB samples measured by XPS (in at.%).

Sample	C	O	S	Si	Zn
rCB	74.0	14.9	2.2	8.4	0.5
rCB_SA_3h	76.4	15.7	3.9	4.0	0.0
G/rCB_SA_3h	69.9	19.8	3.9	6.4	0.0

**Table 4:** Results of the deconvolution of the S2p region, along with the relative atomic percentages of different sulfur species.

Binding Energy [eV]	Assignment	Sample		
		rCB [%]	rCB_SA_3h [%]	G/rCB_SA_3h [%]
163.0–165.5	-C-S-C-	76.9	35.8	37.5
167.5 and 170.5	-C-SO_x_-C-	23.1	64.2	62.5

**Table 5:** Results of the deconvolution of C1s regions together with the relative atomic percentage of different carbon species.

Binding Energy [eV]	Assignment	Sample		
		rCB [%]	rCB_SA_3h [%]	G/rCB_SA_3h [%]
284.5	C-C, C-H, C=C	65.4	69.0	65.3
285.9–286.1	C-O in phenol, alcohols, ethers, C-S	19.8	17.8	18.0
287.2–288.0	C=O in carbonyl and quinone	6.6	3.9	6.4
288.4–289.7	O-C=O in carboxyl, esters, carbonate	3.4	5.7	5.5
>291.0	π-π*	4.8	3.6	4.8

**Table 6:** Comparative analysis of the catalytic performance of carbon materials, including rCB materials, based on literature findings.

Catalyst	Operating Conditions	Catalytic Performance	Ref.
AC-S-18M	a batch reactor Gly:A molar ratio = 1:1, t = 6 h; RT; 2.7 wt.% of catalyst	X_Gly_ = 64%, S_Solketal_ = 98%	[83]
AC-S-18M	a batch reactor Gly:A molar ratio = 1:4, t = 6 h; RT; 2.7 wt.% of catalyst	X_Gly_ = 97%, S_Solketal_ = 96%	[83]
GC-1:2	a batch reactor Gly:A molar ratio = 1:3, t = 2 h; RT; 3 wt.% of catalyst	X_Gly_ = 84%, S_Solketal_ = 90%	[33]
BS 9.2	a batch reactor Gly:P molar ratio = 1:10, t = 2 h; 55 °C; 0.2 wt.% of catalyst	X_Gly_ = 90%, S_Solketal_ = 98%	[19]
SCS1	a batch reactor Gly:A molar ratio = 1:6, t = 2 h; 70 °C; 5 wt.% of catalyst	X_Gly_ = 79%, S_Solketal_ = 76%	[75]
HSCS1	a batch reactor Gly:A molar ratio = 1:6, t = 2 h; 70 °C; 5 wt.% of catalyst	X_Gly_ = 82%, S_Solketal_ = 98%	[75]
rCB_SA_3h	a batch reactor Gly:A molar ratio = 1:1, t = 0.5 h; 40 °C; 1 wt.% of catalyst	X_Gly_ = 92%, S_Solketal_ = 96%	This work
G/rCB_SA_3h	a batch reactor; Gly:A molar ratio = 1:1, t = 0.5 h; 40 °C; 1 wt.% of catalyst	X_Gly_ = 92%, S_Solketal_ = 97%	This work

Gly—glycerol; A—acetone; P—propanone; X_Gly_—glycerol conversion; S_Solketal_—selectivity to solketal; AC-S-18M—activated carbon (AC) prepared by chemical activation of olive stone wastes using 18 M aqueous H_2_SO_4_ under 100 °C for 15 h; GC-1:2—acidic carbon-based catalysts prepared by hydrothermal carbonization of a mixture of glycerin and sulfuric acid under 150 °C for 24 h, where the glycerol/H_2_SO_4_ mass ratio was 1:2; BS 9.2—B refers to bio-oil, S refers to sulfuric acid, and 9.2 is the mass ratio H_2_SO_4_/bio-oil; SCS1—thermally treated sulfonated carbon–silica meso composite; HSCS1—hydrothermally treated sulfonated carbon–silica meso composite; rCB_SA_3h—recovered carbon black from the pyrolysis of waste tires, treated with H_2_SO_4_ at 140 °C for 3 h; G/rCB_SA_3h—glucose-modified recovered carbon black from the pyrolysis of waste tires, treated with H_2_SO_4_ at 140 °C for 3 h.

## Data Availability

Data for this paper, including measurements and fitting results are available at Adam Mickiewicz University Poznań Repository as Recovered carbon black for glycerol acetalization, DOI:10.60629/p08q-0c04.

## Supplementary Materials

The following are available online at https://www.mdpi.com/article/10.3390/molecules29174102/s1, Figure S1: Transmission electron microscopy (TEM) images of initial rCB (a) and selected modified samples: rCB_SA_3h (b), rCB_SA_24h (c), rCB_BDS_20h (d); Figure S2: The effect of reaction temperature and reaction time on glycerol conversion and selectivity to solketal obtained on rCB_SA_3h catalyst; Figure S3: FTIR spectra of initial and modified rCB samples after the reaction cycle (spent catalysts); Figure S4: The XPS survey spectra of spent rCB samples; Figure S5: High-resolution XPS C1s spectra of spent rCB samples; Figure S6: High-resolution XPS S2p spectra of spent rCB samples; Table S1: Elemental composition of the initial and spent rCB samples measured by XPS (in at.%); Table S2: Results of the deconvolution of C1s regions together with the relative atomic percentage of different carbon species; Table S3: Results of the deconvolution of the S 2p region, along with the relative atomic percentages of different sulfur species. Scheme S1: Pathway of the acetylation reaction of glycerol with acetone; Scheme S2. The proposed mechanism for glycerol acetalization reaction with acetone over modified rCB catalysts.

## References

[1] Sofi A. (2018). Effect of Waste Tyre Rubber on Mechanical and Durability Properties of Concrete—A Review. ASEJ.

[2] Sienkiewicz M., Kucinska-Lipka J., Janik H., Balas A. (2012). Progress in Used Tyres Management in the European Union: A Review. Waste Manag..

[3] Zerin N.H., Rasul M.G., Jahirul M.I., Sayem A.S.M. (2023). End-of-life Tyre Conversion to Energy: A Review on Pyrolysis and Activated Carbon Production Processes and Their Challenges. Sci. Total Environ..

[4] Giugliano M., Cernuschi S., Ghezzi U., Grosso M. (1999). Experimental Evaluation of Waste Tires Utilization in Cement Kilns. JAWMA.

[5] Jahirul M.I., Hossain F.M., Rasul M.G., Chowdhury A.A. (2021). A Review on the Thermochemical Recycling of Waste Tyres to Oil for Automobile Engine Application. Energies.

[6] Martínez J.D., Puy N., Murillo R., García T., Navarro M.V., Mastral A.M. (2013). Waste Tyre Pyrolysis—A Review. Renew. Sustain. Energy Rev..

[7] Arabiourrutia M., Lopez G., Artetxe M., Alvarez J., Bilbao J., Olazar M. (2020). Waste Tyre Valorization by Catalytic Pyrolysis—A Review. Renew. Sustain. Energy Rev..

[8] Urrego-Yepes W., Cardona-Uribe N., Vargas-Isaza C.A., Martínez J.D. (2021). Incorporating the recovered carbon black produced in an industrial-scale waste tire pyrolysis plant into a natural rubber formulation. J. Environ. Manag..

[9] Chan O.S., Cheung W.H., McKay G. (2012). Single and Multicomponent Acid Dye Adsorption Equilibrium Studies on Tyre Demineralised Activated Carbon. Chem. Eng. J..

[10] Antoniou N., Stavropoulos G., Zabaniotou A. (2014). Activation of End of Life Tyres Pyrolytic Char for Enhancing Viability of Pyrolysis—Critical Review, Analysis and Recommendations for a Hybrid Dual System. Renew. Sustain. Energy Rev..

[11] Chaichana E., Wiwatthanodom W., Jongsomjit B. (2019). Carbon-based Catalyst from Pyrolysis of Waste Tire for Catalytic Ethanol Dehydration to Ethylene and Diethyl Ether. Int. J. Chem. Eng..

[12] Dell’Era A., Pasquali M., Tarquini G., Scaramuzzo F.A., De Gasperis P., Prosini P.P., Mezzi A., Tuffi R., Cafiero L. (2018). Carbon Powder Material Obtained from an Innovative High Pressure Water Jet Recycling Process of Tires Used as Anode in Alkali Ion (Li, Na) Batteries. Solid State Ion..

[13] Zhang W., Li S., Zhou A., Song H., Cui Z., Du L. (2021). Recent Advances and Perspectives in Lithium−Sulfur Pouch Cells. Molecules.

[14] Djuandhi L., Gaikwad V., Cowie B.C.C., Sahajwalla V., Sharma N. (2021). Repurposing Waste Tires as Tunable Frameworks for Use in Sodium-ion and Lithium−sulfur Batteries. ACS Sustain. Chem. Eng..

[15] Husár J., Haydary J., Šuhaj P. (2019). Potential of Tire Pyrolysis Char as Tar-cracking Catalyst in Solid Waste and Biomass Gasification. Chem. Pap..

[16] Krasnovskikh M., Mokrushin I., Novoselov K., Kulikova Y., Toderaş M., Bassyouni M., Babich O. (2024). Recovered Carbon Black from Tires as Carbon Carrier in Metal Oxide Catalytic Systems. S. Afr. J. Chem. Eng..

[17] Corrêa I., Faria R.P.V., Rodrigues A.E. (2021). Continuous Valorization of Glycerol into Solketal: Recent Advances on Catalysts, Processes, and Industrial Perspectives. Sustain. Chem..

[18] Mo X., López D.E., Suwannakarn K., Liu Y., Lotero E., Goodwin J.G., Lu C. (2008). Activation and Deactivation Characteristics of Sulfonated Carbon Catalysts. J. Catal..

[19] Ballotin F.C., da Silva M.J., Teixeira A.P.d.C., Lago R.M. (2020). Amphiphilic Acid Carbon Catalysts Produced by Bio-oil Sulfonation for Solvent-free Glycerol Ketalization. Fuel.

[20] Zhang B., Gao M., Geng J., Cheng Y., Wang X., Wu C., Wang Q., Liu S., Cheung S.M. (2021). Catalytic Performance and Deactivation Mechanism of a One-step Sulfonated Carbon-based Solid-acid Catalyst in an Esterification Reaction. Renew. Energy.

[21] Zailan Z., Tahir M., Jusoh M., Zakaria Z.Y. (2021). A Review of Sulfonic Group Bearing Porous Carbon Catalyst for Biodiesel Production. Renew. Energy.

[22] Ayoob A.K., Fadhil A.B. (2020). Valorization of Waste Tires in the Synthesis of an Effective Carbon Based Catalyst for Biodiesel Production from a Mixture of Non-edible Oils. Fuel.

[23] Malaika A., Kowalska-Kuś J., Końska K., Ptaszyńska K., Jankowska A., Held A., Wróblewski K., Kozłowski M. (2023). Upgrading Pyrolytic Residue from End-of-Life Tires to Efficient Heterogeneous Catalysts for the Conversion of Glycerol to Acetins. Molecules.

[24] Gebremariam S.N., Marchetti J.M. (2018). Economics of biodiesel production: Review. Energy Convers. Manag..

[25] Bohlouli A., Mahdavian L. (2019). Catalysts used in biodiesel production: A review. Biofuels.

[26] Wang Q., Xie W., Guo L. (2022). Molybdenum and zirconium oxides supported on KIT-6 silica: A recyclable composite catalyst for one–pot biodiesel production from simulated low-quality oils. Renew. Energy.

[27] Hou S., Xie W. (2024). Three-dimensional hierarchical meso/macroporous Mo/Ce/TiO_2_ composites enhances biodiesel production from acidic soybean oil by transesterification-esterifiications. Energy Convers. Manag..

[28] Xie W., Wang H. (2021). Grafting copolymerization of dual acidic ionic liquid on core-shell structured magnetic silica: A magnetically recyclable Brönsted acid catalyst for biodiesel production by one-pot transformation of low-quality oils. Fuel.

[29] Kowalska-Kuś J., Held A., Nowińska K. (2020). A Continuous-flow Process for the Acetalization of Crude Glycerol with Acetone on Zeolite Catalysts. J. Chem. Eng..

[30] Kaura J., Sarma A.K., Jha M.K., Gera P. (2020). Valorisation of crude glycerol to value-added products: Perspectives of process technology, economics and environmental issues. Biotechnol. Rep..

[31] Checa M., Nogales-Delgado S., Montes V., Encinar J.M. (2020). Recent Advances in Glycerol Catalytic Valorization: A Review. Catalysts.

[32] Vannucci J.A., Gatti M.N., Cardaci N., Nichio N.N. (2022). Economic Feasibility of a Solketal Production Process from Glycerol at Small Industrial Scale. Renew. Energy.

[33] Gonçalves M., Rodrigues R., Galhardo T.S., Carvalho W.A. (2016). Highly Selective Acetalization of Glycerol with Acetone to Solketal over Acidic Carbon-based Catalysts from Biodiesel Waste. Fuel.

[34] García E., Laca M., Pérez E., Garrido A., Peinado J. (2008). New Class of Acetal Derived from Glycerin as a Biodiesel Fuel Component. Energy Fuels.

[35] García H., García J.I.C.O., Fraile J.M.C.O., Mayoral J.A. (2008). Solketal: Green and Catalytic Synthesis and Its Classification as a Solvent—2,2-dimethyl-4-hidroxymethyl-1,3-dioxolane, an Interesting Green Solvent Produced Through Heterogeneous Catalysis. Chem. Today.

[36] Deutsch J., Martin A., Lieske H. (2007). Investigations on Heterogeneously Catalysed Condensations of Glycerol to Cyclic Acetals. J. Catal..

[37] Vicente G., Melero J.A., Morales G., Paniagua M., Martín E. (2010). Acetalisation of Bio-glycerol with Acetone to Produce Solketal over Sulfonic Mesostructured Silicas. Green Chem..

[38] Janiszewska E., Kowalska-Kuś J., Góra-Marek K., Szymocha A., Nowińska K., Kowalak S. (2019). Modification of Silicalite-1 with Ammonium Compounds Aimed at Preparation of Acidic Catalyst for Acetalization of Glycerol with Acetone. Appl. Catal. A Gen..

[39] Ferreira P., Fonseca I.M., Ramos A.M., Vital J., Castanheiro J.E. (2010). Valorisation of Glycerol by Condensation with Acetone over Silica-included Heteropolyacids. App. Catal. B Environ..

[40] Kowalska-Kuś J., Held A., Frankowski M., Nowinska K. (2017). Solketal Formation from Glycerol and Acetone over Hierarchical Zeolites of Different Structure as Catalysts. J. Mol. Catal. A Chem..

[41] Kowalska-Kuś J., Held A., Nowińska K. (2020). Solketal Formation in a Continuous Flow Process over Hierarchical Zeolites. ChemCatChem.

[42] Nair G.S., Adrijanto E., Alsalme A., Kozhevnikov I.V., Cooke D.J., Brown D.R., Shiju N.R. (2012). Glycerol utilization: Sol-vent-free acetalisation over niobia catalysts. Catal. Sci. Technol..

[43] Stawicka K., Díaz-Álvarez A.E., Calvino-Casilda V., Trejda M., Bañares M.A., Ziolek M. (2016). The role of Brønsted and Lewis acid sites in acetalization of glycerol over modified mesoporous cellular foams. J. Phys. Chem. C.

[44] Fan C.-N., Xu C.-H., Liu C.-Q., Huang Z.-Y., Liu J.-Y., Ye Z.-X. (2012). Catalytic acetalization of biomass glycerol with acetone over TiO_2_–SiO_2_ mixed oxides. React. Kinet. Mech. Catal..

[45] da Silva C.X.A., Gonalves V.L.C., Mota C.J.A. (2009). Review Article Recent Advances in the Valorization of Biodiesel By-Product Glycerol to Solketal. Green Chem..

[46] Nakajima K., Hara M. (2012). Amorphous Carbon with SO_3_H Groups as a Solid Brønsted Acid Catalyst. ACS Catal..

[47] Thommes M., Kaneko K., Neimark A.V., Olivier J.P., Rodriguez-Reinoso F., Rouquerol J., Sing K.S.W. (2015). Physisorption of gases, with special reference to the evaluation of surface area and pore size distribution (IUPAC Technical Report). Pure Appl. Chem..

[48] Geng L., Yu G., Wang Y., Zhu Y. (2012). Ph-SO_3_H-modified mesoporous carbon as an efficient catalyst for the esterification of oleic acid. Appl. Catal. A Gen..

[49] Zhang X., Wang T., Ma L., Chang J. (2008). Vacuum pyrolysis of waste tires with basic additives. Waste Manag..

[50] Li S.-Q., Yao Q., Chi Y., Yan J.H., Chen K.F. (2004). Pilot-scale pyrolysis of scrap tires in a continuous rotary kiln reactor. Ind. Eng. Chem. Res..

[51] Fraile J.M., García-Bordejé E., Pire E., Roldán L. (2014). New insights into the strength and accessibility of acid sites of sulfonated hydrothermal carbon. Carbon.

[52] Ptaszyńska K., Malaika A., Morawa Eblagon K., Figueiredo J.L., Kozłowski M. (2024). Promoting Effect of Ball Milling on the Functionalization and Catalytic Performance of Carbon Nanotubes in Glycerol Etherification. Molecules.

[53] Mis-Fernandez R., Azamar-Barrios J.A., Rios-Soberanis C.R. (2008). Characterization of the powder obtained from wasted tires reduced by pyrolysis and thermal shock process. J. Appl. Res. Technol..

[54] Ibrahim R.M., Markom M., Abdullah H. (2015). Optical Properties of Ni^2+^-, Co^2+^-, and Mn^2+^-doped ZnS Nanoparticles Synthesized Using Reverse Micelle Method. J. Solid State Sci. Techn..

[55] Hu H., Fang Y., Liu H., Yu R., Luo G., Liu W., Li A., Yao H. (2014). The fate of sulfur during rapid pyrolysis of scrap tires. Chemosphere.

[56] Tang H., Hu H., Li A., Yi B., Li X., Yao D., Yao H., Yuan H. (2021). Removal of impurities from waste tire pyrolysis char using the molten salt thermal treatment. Fuel.

[57] Larcheri S., Armellini C., Rocca F., Kuzmin A., Kalendarev R., Dalba G., Graziola R., Purans J., Pailharey D., Jandard F. (2006). X-ray studies on optical and structural properties of ZnO nanostructured thin films. Superlattices Microstruct..

[58] Kang G.S., Lee G., Youn S., Cho S.Y., Joh H.I., Lee D.C., Lee S. (2021). Recycling of waste tires by synthesizing N-doped carbon-based catalysts for oxygen reduction reaction. Appl. Surf. Sci..

[59] López F.A., Centeno T.A., Rodríguez O., Alguacil F.J. (2013). Preparation and characterization of activated carbon from the char produced in the thermolysis of granulated scrap tyres. J. Air Waste Manag. Assoc..

[60] Guo Y., Zeng Z., Li Y., Huang Z., Cui Y. (2018). In-situ sulfur-doped carbon as a metal-free catalyst for persulfate activated oxidation of aqueous organics. Catal. Today.

[61] Wang Z., Li P., Chen Y., He J., Zhang W., Schmid O.G., Li Y. (2014). Pure thiophene–sulfur doped reduced graphene oxide: Synthesis, structure, and electrical properties. Nanoscale.

[62] Sevilla M., Fuertes A.B. (2012). Highly porous S-doped carbons. Micropor. Mesopor. Mater..

[63] Zhang L., Wang Y., Wan K., Piaoa J.-H., Liang Z.-X. (2018). Effective sulfur-doping in carbon by high-temperature molten salt bath and its electrocatalysis for oxygen reduction reaction. Electrochem. Commun..

[64] Wu H., Sui X., Lei Y., Liu L., Xu W., Liang G., Li C., Li X. (2024). Compositing of Co_3_O_4_ with boron nitride to promote the catalytic performance for methane oxidation. Fuel.

[65] Malaika A., Ptaszyńska K., Morawa Eblagon K., Pereira M.F.R., Figueiredo J.L., Kozłowski M. (2021). Solid acid carbon catalysts for sustainable production of biofuel enhancers via transesterification of glycerol with ethyl acetate. Fuel.

[66] Morgan D.J. (2021). Comments on the XPS Analysis of Carbon Materials. C.

[67] Suganuma S., Nakajima K., Kitano M., Yamaguchi D., Kato H., Hayashi S., Hara M. (2008). Hydrolysis of Cellulose by Amorphous Carbon Bearing SO_3_H. J. Am. Chem. Soc..

[68] Zhao W., Yang B., Yi C., Lei Z., Xu J. (2010). Etherification of Glycerol with Isobutylene to Produce Oxygenate Additive Using Sulfonated Peanut Shell Catalyst. Ind. Eng. Chem. Res..

[69] Ali U.F., Hussin F., Gopinath S.C.B., Aroua M.K., Khamidun M.H., Jusoh N., Ibrahim N., Ahmad S.F.K. (2022). Advancement in recycling waste tire activated carbon to potential adsorbents. Environ. Eng. Res..

[70] Rechnia P., Maliaka A., Kozłowski M. (2015). Synthesis of tert-amyl methyl ether (TAME) over modified activated carbon catalysts. Fuel.

[71] Corrêa A., Bastos R., Filho G., Zamian J., Conceição L. (2020). Preparation of sulfonated carbon-based catalysts from murumuru kernel shell and their performance in the esterifcation reaction. RSC Adv..

[72] Choi D.S., Yoo S.H., Lee S. (2019). Safer and more effective route for polyethylene-derived carbon fiber fabrication using electron beam irradiation. Carbon.

[73] Malaika A., Heinrich M., Goscianska J., Kozłowski M. (2020). Synergistic effect of functional groups in carbonaceous spheres on the formation of fuel enhancers from glycerol. Fuel.

[74] Domínguez-Barroso V., Herrera C., Ángeles Larrubia M., González-Gil R., Cortés-Reyes M., Alemany L.J. (2019). Continuous-Flow Process for Glycerol Conversion to Solketal Using a Brönsted Acid Functionalized Carbon-Based Catalyst. Catalysts.

[75] Nandan D., Sreenivasulu P., Sivakumar Konathala L.N., Kumar M., Viswanadham N. (2013). Acid functionalized carbon–silica composite and its application for solketal production. Microporous Mesoporous Mater..

[76] Fernández P., Fraile J.M., García-Bordejé E., Pires E. (2019). Sulfonated hydrothermal carbons from cellulose and glucose as catalysts for glycerol ketalization. Catalysts.

[77] Ferreira P., Fonseca I.M., Ramos A.M., Vital J., Castanheiro J.E. (2011). Acetylation of glycerol over heteropolyacids supported on activated carbon. Catal. Commun..

[78] Konwar L.J., Boro J., Deka D. (2014). Review on latest developments in biodiesel production using carbon-based catalysts. Renew. Sustain. Energy Rev..

[79] Rechnia-Gorący P., Malaika A., Kozłowski M. (2018). Acidic activated carbons as catalysts of biodiesel formation. Diam. Relat. Mater..

[80] Hosseini M.-S., Masteri-Farahani M., Ghahremani M., Forouzeshfar N. (2021). New approach for sulfonation of carbonaceous materials: Highly efficient solid acid catalysts for benzaldehyde acetalization with ethylene glycol. J. Phys. Chem. Solids.

[81] Pan H., Sun J., Liu J., Zhang Y., Zhou S. (2021). Preparation of sulfonated carbon derived from orange peel and its application in esterification. Chem. Phys. Lett..

[82] Mo X., Lotero E., Lu C., Liu Y., Goodwin J.G. (2008). A Novel Sulfonated Carbon Composite Solid Acid Catalyst for Biodiesel Synthesis. Catal. Lett..

[83] Rodrigues R., Gonçalves M., Mandelli D., Percarmona P.P., Carvalho W.A. (2014). Solvent-free conversion of glycerol to solketal catalysed by activated carbons functionalised with acid groups. Catal. Sci. Technol..

[84] https://contec.tech/product/recovered-carbon-black/.

