# Supplementary material for: Synthesis of Solketal Catalyzed by Acid-Modified Pyrolytic Carbon Black from Waste Tires
Source: Molecules. 2024 Aug 29;29(17):4102. doi: 10.3390/molecules29174102 (PMC11397316; doi:10.3390/molecules29174102)
Supplement: Supplementary file 1 [file molecules-29-04102-s001.zip › molecules-3155766-supplementary.pdf]

Synthesis of solketal catalyzed by acid modified pyrolytic carbon black from waste tires

Jolanta Kowalska-Kuś[1*], Anna Malaika[1], Agnieszka Held[1], Aldona Jankowska[1], Ewa Janiszewska[1], Michał Zieliński[1], Krystyna Nowińska[1], Stanisław Kowalak[1*], Klaudia Końska[2], Krzysztof Wróblewski[2]

[1] Faculty of Chemistry, Adam Mickiewicz University, Uniwersytetu Poznańskiego 8, 61-614 Poznań, Poland; anna.malaika@amu.edu.pl (A.M.); awaclaw@amu.edu.pl (A.H.), aljan@amu.edu.pl (A.J.); eszym@amu.edu.pl (E.J.); mardok@amu.edu.pl (M.Z.), krysnow@amu.edu.pl (K.N.);
[2] Contec, al. Jerozolimskie 142A, 02-305 Warszawa; k.konska@contec.tech (K.K.); k.wroblewski@contec.tech(K.W.)
* Correspondence: jolakow@amu.edu.pl (J.K.-K.), skowalak@amu.edu.pl (S.K.)

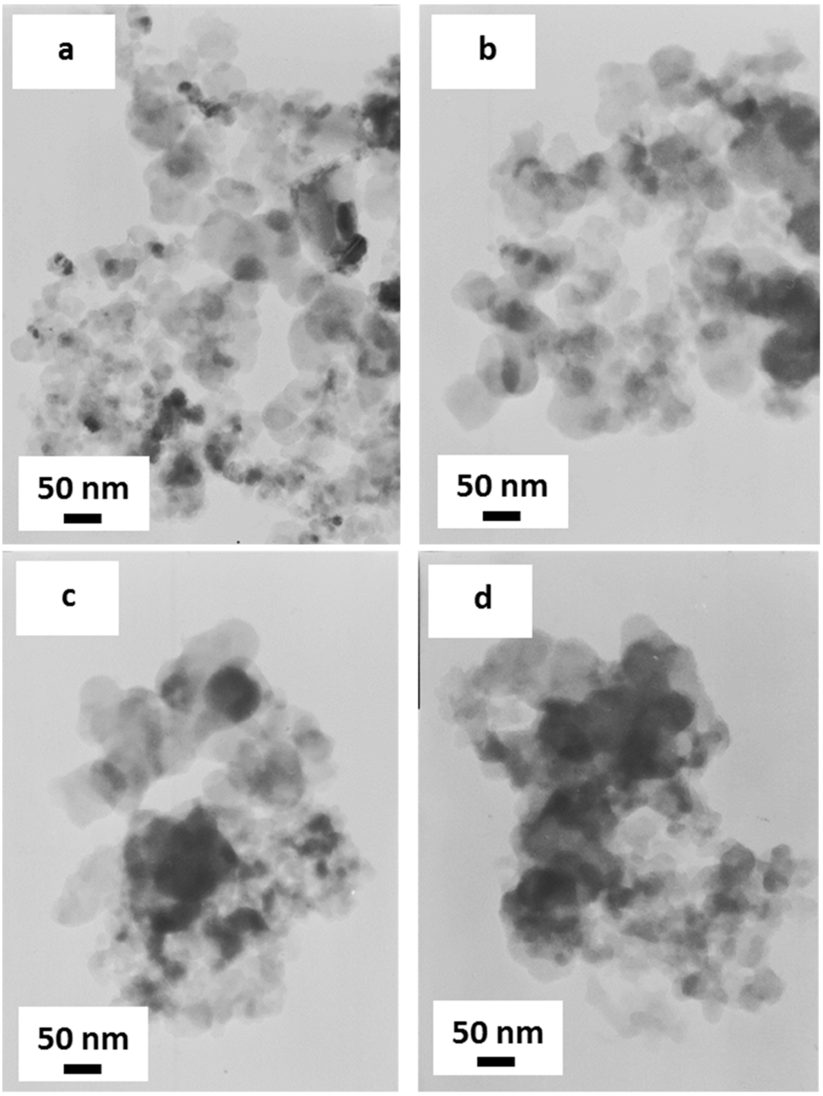

Figure S1. Transmission electron microscope (TEM) images of initial rCB (a) and selected modified samples: rCB_SA_3h (b), rCB_SA_24h (c), rCB_BDS_20h (d).

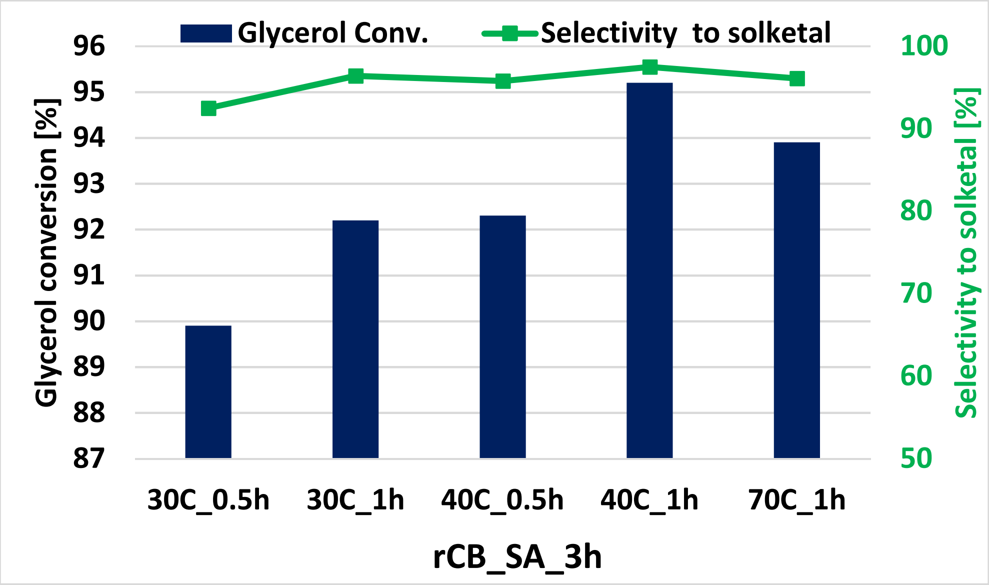

Figure S2. The effect of reaction temperature and reaction time on glycerol conversion and selectivity to solketal obtained on rCB_SA_3h catalyst.

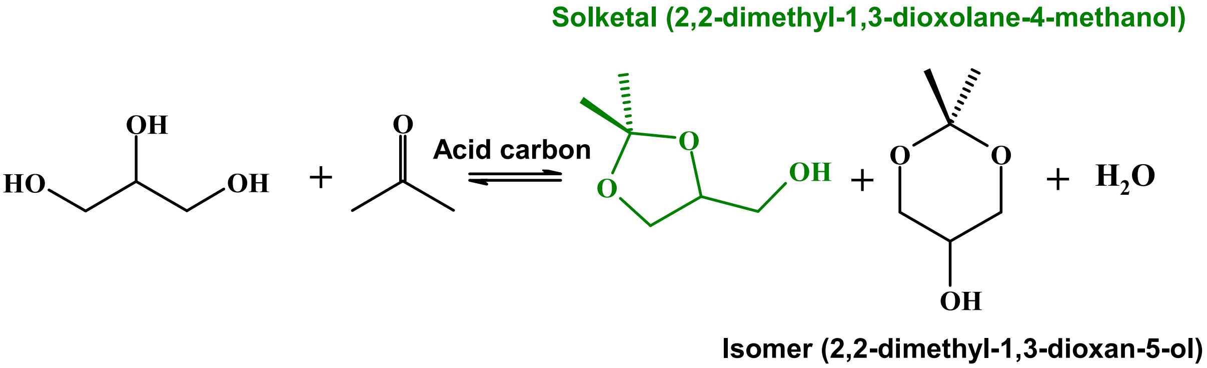

Scheme S1. Pathway of the acetalization reaction of glycerol with acetone.

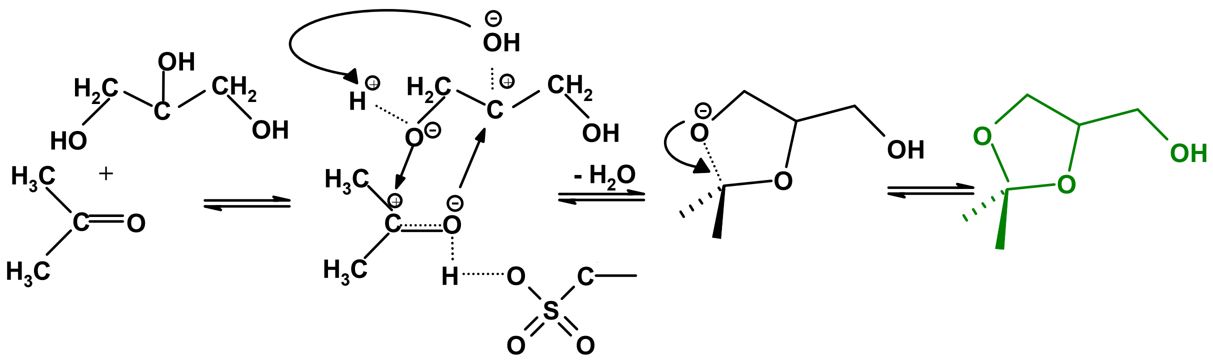

Scheme S2. The proposed mechanism for glycerol acetalization reaction with acetone over sulfonated rCB catalysts.

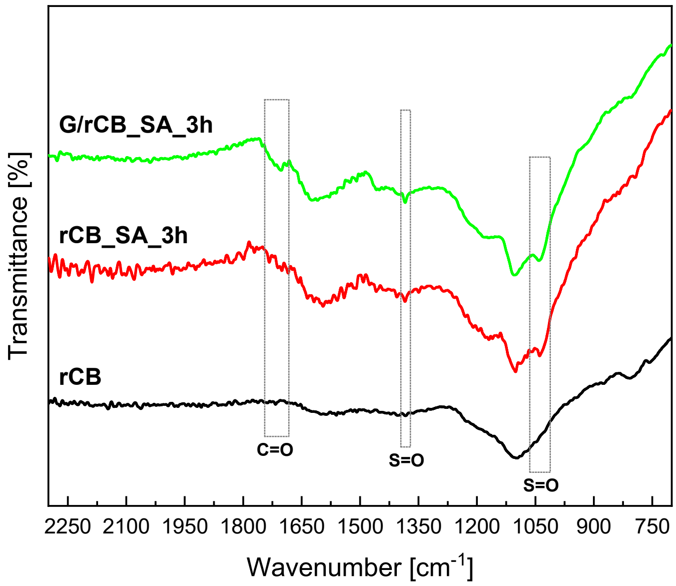

Figure S3. FT-IR spectra of initial and modified rCB samples after the 1st reaction cycle (spent catalysts).

Table S1. Elemental composition of the initial and spent rCB samples measured by XPS (in at.%).

Sample	C	O	S	Si	Zn
rCB_SA_3h	76.4	15.7	3.9	4.0	0.0
rCB_SA_3h_spent	75.0	17.9	2.4	4.7	0.0
G/rCB_SA-3h	69.9	19.8	3.9	6.4	0.0
G/rCB_SA-3h_spent	71.0	20.9	1.8	6.3	0.0

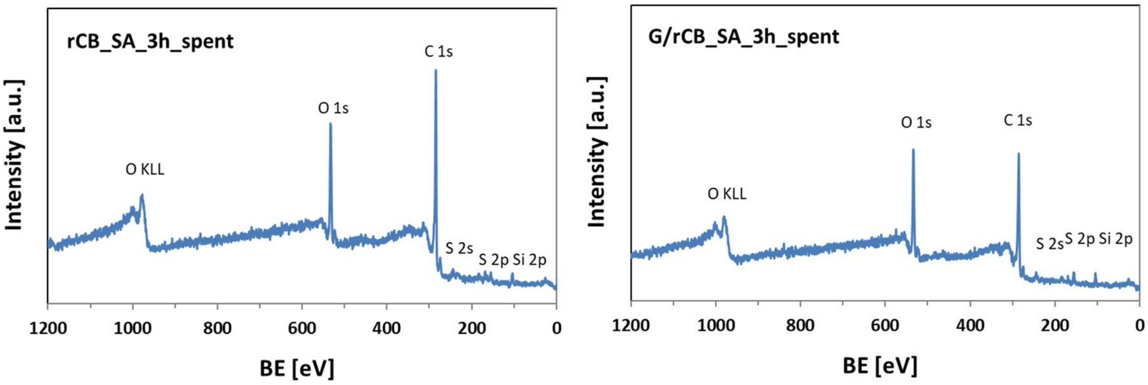

Figure S4. The XPS survey spectra of spent rCB samples.

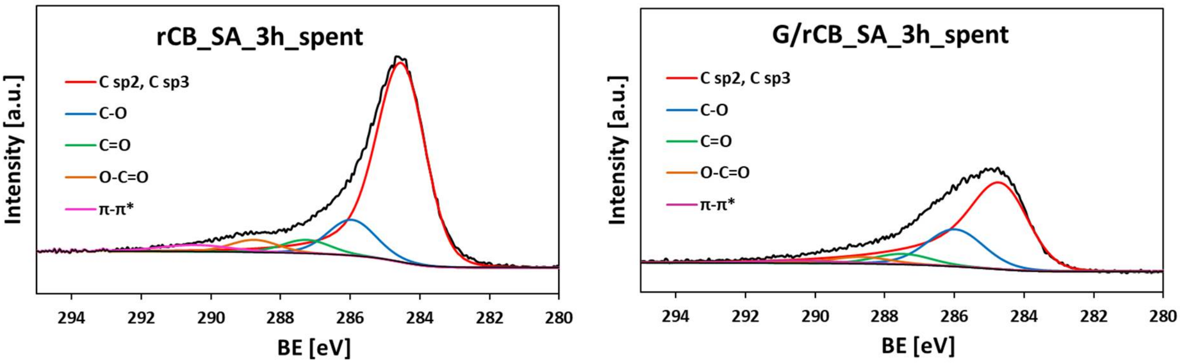

Figure S5. High-resolution XPS C1s spectra of spent rCB samples.

Table S2. Results of the deconvolution of C1s regions together with the relative atomic percentage of different carbon species.

Binding Energy [eV]	Assignment	Sample			
		rCB_SA_3h [%]	rCB_SA_3h spent [%]	G/rCB_SA_3h [%]	G/rCB_SA_3h spent [%]
284.5	C-C, C-H, C=C	74.7	75.5	71.5	66.2
285.9-286.1	C-O in phenol, alcohols, ethers, C-S	13.8	12.3	13.4	21.6
287.2-288.0	C = O in carbonyl and quinone	3.1	4.6	5.5	6.4
288.4-289.7	O-C = O in carboxyl, esters, carbonate	5.1	4.3	4.9	3.9
>291.0	π-π*	3.3	3.3	4.7	1.9

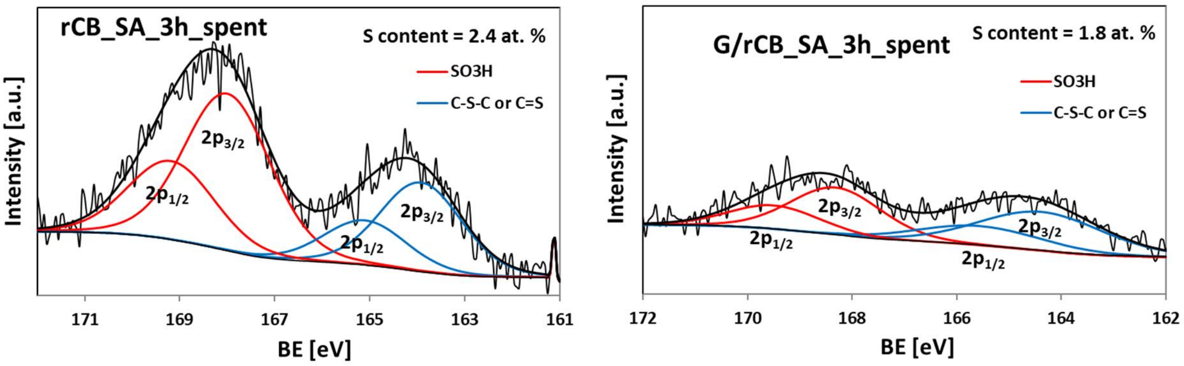

Figure S6. High resolution XPS S2p spectra of spent rCB samples.

Table S3. Results of the deconvolution of the S 2p region, along with the relative atomic percentages of different sulfur species.

Binding Energy [eV]	Assignment	Sample			
		rCB_SA_3h [%]	rCB_SA_3h spent [%]	G/rCB_SA_3h [%]	G/rCB_SA_3h spent [%]
163.0-165.5	-C-S-C-	35.8	35.1	37.5	52.6
167.5 and 170.5	-C-SO_x-C-	64.2	64.9	62.5	47.4
